# Data Fusion Architectures for Orthogonal Redundant Inertial Measurement Units

**DOI:** 10.3390/s18061910

**Published:** 2018-06-12

**Authors:** Eric Gagnon, Alexandre Vachon, Yanick Beaudoin

**Affiliations:** 1 Defence Research and Development Canada, Quebec, QC G3J 1X5, Canada; 2 Numérica Technologies Inc., Quebec, QC G2E 4P8, Canada; Alexandre.Vachon.NUMERICA@drdc-rddc.gc.ca; 3 Département de génie électrique et génie informatique, Université Laval, Quebec, QC G1V 0A6, Canada; yanick.beaudoin.1@ulaval.ca

**Keywords:** orthogonal redundant inertial measurement units, data fusion architectures, sensors bias

## Abstract

This work looks at the exploitation of large numbers of orthogonal redundant inertial measurement units. Specifically, the paper analyses centralized and distributed architectures in the context of data fusion algorithms for those sensors. For both architectures, data fusion algorithms based on Kalman filter are developed. Some of those algorithms consider sensors location, whereas the others do not, but all estimate the sensors bias. A fault detection algorithm, based on residual analysis, is also proposed. Monte-Carlo simulations show better performance for the centralized architecture with an algorithm considering sensors location. Due to a better estimation of the sensors bias, the latter provides the most precise and accurate estimates and the best fault detection. However, it requires a much longer computational time. An analysis of the sensors bias correlation is also done. Based on the simulations, the biases correlation has a small effect on the attitude rate estimation, but a very significant one on the acceleration estimation.

## 1. Introduction

In the beginning of the 90s, a novel sensor design technique changed the manufacturing and operation of inertial navigation systems (INS). Micro-electro-mechanical systems (MEMS) integrate the classical mechanical design in the integrated circuit fabrication technology. This new class of small inertial measurement units (IMUs) can then be fabricated in large batches, drastically reducing the production costs. However, their smaller size is problematic; a smaller sensor is more sensitive to temperature and has a smaller scale factor, resulting in a lower signal-to-noise ratio than classical IMUs. On the other hand, the MEMS’ small size and low cost make them suitable to build arrays of redundant sensors for fused measurements objective. The combination of inertial sensors in arrays has therefore become an important field of research lately [1].

The most common purpose for redundant inertial measurement units (RIMUs) in navigation systems is to facilitate the detection and isolation of faulty sensors [2]. The precision and accuracy improvement provided by using multiple measurements is often a secondary objective. In this optic, the studied sensors are, more than often, positioned in a non-orthogonal skewed configuration, known as SRIMU, or on regular polyhedrons. In a SRIMU, the sensors are distributed on a cone, in a way that there is a constant angle between two consecutive sensors [3]. This configuration provides the maximum redundancy and, hence, encapsulates the maximum amount of information for a specific number of IMUs [4]. The regular polyhedrons are, on their parts, the optimal configuration for navigation and fault detection and isolation (FDI) [5].

However, these configurations are interesting only for a relatively low number of redundancies and for costly sensors [6]. When dealing with a large number of IMUs, these optimal configurations are very complex and the interest of lowering the cost of the redundancy by using the minimal number of IMUs is overshadowed by the cost due to the complexity of the assembly. In this case, an orthogonal configuration, where triads of sensors are mounted parallel one to the others, can be interesting. This configuration is much easier to assemble, but, in comparison to the SRIMU and polyhedrons configurations, has poor FDI performance [6]. In order to provide the same level of robustness (same number of detected faults), a higher number of IMUs is required in the orthogonal configuration. However, for RIMU based on MEMS, which are relatively cheap and small, this is not an issue. As a result, the orthogonal configuration should be seriously considered for those sensors [6].

The handling of these redundant measurements can be categorised in two types: centralized or distributed. In the centralized architecture, all sensor measurements are directly processed by a single central data fusion filter in order to obtain the state estimates. This architecture is the optimal [7] and most common design in current INS [8], even though it is not necessarily the most efficient one. With multiple sensors of different types, the centralized filter computation might be time-consuming [8], and it is not the ideal method for a fault tolerant multi-sensors scheme [9]. However, when only IMUs are used, the centralized architecture remains time-consuming, but does not exhibit FDI sub-performance [2].

On its part, the distributed architecture is a multi-stage architecture where parallel local filters independently process the measurements of their corresponding sensors and their outputs are used by a master data fusion filter which produces the final state estimates. Also, the master filter can send back information to the local filters in order to reset some components of their internal state. As the centralized architecture, this architecture can offer optimal performance, but only when all filters model the full state and when they are all ran at the same rate [7]. The main advantage of this architecture is its better FDI performance [9]. This architecture has been applied to multiple types of multi-sensors navigation systems [9,10] and to multiple IMUs systems [2,4].

For vehicle navigation, the fusion of IMUs is mainly studied as part of navigation algorithm aided by Global Positioning System (GPS). However, some works [11,12,13] discard the GPS and rely on alternative (radar or vision) measurement devices, while others rely purely on inertial sensors, accelerometers only [14,15] or accelerometers and gyroscopes [16].

Considering the fictitious forces due to sensors location is not common in RIMU data fusion algorithms, but has been done [2,16]. Similarly, estimating the sensors bias within the algorithms has been considered [17,18,19]. It is also the case for the analysis of correlation between the sensor random walk noises [20,21], but not a correlation between the biases of the sensors.

This paper intends to simultaneously address those three aspects (sensors locations, biases estimation and biases correlation) within navigation based on IMUs only. Above all, the paper studies their effects on each type of data fusion architecture. The main objective is the identification of the ideal architecture for data fusion of a large number of orthogonal RIMU. For the two studied architectures, data fusion algorithms based on Kalman filter are used (Section 2). Those algorithms, notably, estimate the sensors bias. For both architectures, an algorithm which considers sensors location, and one which does not, are proposed. A baseline algorithm, averaging the measurements, is used as a comparison point. A fault detection and isolation algorithm, based on residual statistical analysis, is also presented (Section 3). Those algorithms are then compared using Monte-Carlo simulations (Section 4). The simulated application consists in an artillery shell (spin stabilized projectile) equipped with strapdown orthogonal RIMU having, as a first case, uncorrelated biases and, as a second case, correlated ones. A simulations-based analysis is mandatory for this application; it would have been impossible to test the same amount of sensors locations, noises, biases and faults in the field.

## 2. Data Fusion Algorithms

This section proposes data fusion algorithms based on Kalman filter for MEMS RIMU. MEMS sensors are characterized by large random walk and bias instability [22]. The random walk is, by nature, a white noise which can be efficiently processed by a Kalman filter, but the bias instability is a second order random walk [22] and requires a specific treatment in order to be estimated by a Kalman filter.

Obviously, it is impossible, even with MEMS, to have perfectly collocated sensors. The distance between the IMUs can be very small and much smaller than the sensitivity of the sensors, but this distance still influence the quality of the estimations. The Grubin transformation [23], which expresses the fictitious forces applied on a point not located at the centre of mass of a body, is used to include the effect of the angular motion on the accelerometer measurements by taking into account the sensors location in the models. A triad of accelerometers, located at the sensor position $\left(s_{sb}\right)$, then measures three accelerations: the nominal linear acceleration (a), the angular acceleration $\left(\dot{\omega }\times s_{sb}\right)$ and the centrifugal acceleration $\left(\omega \times \left(\omega \times s_{sb}\right)\right)$. On its part, the gyroscopes triad is not affected by its location and still measures the angular velocity (ω) only.

For the centralized and distributed architectures, the Kalman filter prediction model required to consider the sensors location will be presented. Then, by eliminating the fictitious forces, a model for an algorithm not considering sensors location will be derived. Similarly, for the baseline architecture, the algorithm discarding sensors location is derived from the algorithm considering sensors location.

### 2.1. Centralized Architecture

In a typical GPS/INS navigation solution, the coupling between the acceleration and attitude rate measurements appears, through the rotation matrix, in the velocity and position vectors [24]. However, without GPS receiver, the velocity, position and attitude are unobservable, and can, without performance degradation, be eliminated from the data fusion algorithm in order to decrease its complexity. For the centralized architecture, the filter state is then composed of the acceleration estimates $\left(a_{e}\right)$, the attitude rate estimates $\left(\omega_{e}\right)$ and the biases of all accelerometers $\left(b_{a}\right)$ and of all gyroscopes $\left(b_{\omega }\right)$:

(1) $$x={\left[\begin{array}{cccccccc} a_{e} & {b_{a}}_{1} & \cdots & {b_{a}}_{n} & \omega_{e} & {b_{\omega }}_{1} & \cdots & {b_{\omega }}_{n} \end{array}\right]}^{T}$$

On its part, the state propagation equation, which is linear, is:

(2) $$x_{k+1}={\left[\begin{array}{c} a_{e}\\ \begin{array}{c} {b_{a}}_{1}\\ \vdots \\ {b_{a}}_{n}\\ \omega_{e}\;\\ {b_{\omega }}_{1}\\ \vdots \\ {b_{\omega }}_{n} \end{array} \end{array}\right]}_{k+1}=\left[\begin{array}{cccccccc} I_{3} & 0_{3} & \cdots & 0_{3} & 0_{3} & 0_{3} & \cdots & 0_{3}\\ 0_{3} & I_{3} & \cdots & 0_{3} & 0_{3} & 0_{3} & \cdots & 0_{3}\\ \vdots & \vdots & \ddots & \vdots & \vdots & \vdots & \ddots & \vdots \\ 0_{3} & 0_{3} & \cdots & I_{3} & 0_{3} & 0_{3} & \cdots & 0_{3}\\ 0_{3} & 0_{3} & \cdots & 0_{3} & I_{3} & 0_{3} & \cdots & 0_{3}\\ 0_{3} & 0_{3} & \cdots & 0_{3} & 0_{3} & I_{3} & \cdots & 0_{3}\\ \vdots & \vdots & \ddots & \vdots & \vdots & \vdots & \ddots & \vdots \\ 0_{3} & 0_{3} & \cdots & 0_{3} & 0_{3} & 0_{3} & \cdots & I_{3} \end{array}\right]x_{k}+{\left[\begin{array}{c} \begin{array}{c} v_{a}\\ {v_{ba}}_{1} \end{array}\\ \begin{array}{c} \vdots \\ {v_{ba}}_{n}\\ v_{\omega }\\ {v_{b\omega }}_{1}\\ \vdots \end{array}\\ {v_{b\omega }}_{n} \end{array}\right]}_{k}$$

and the nonlinear output equation is:

(3) $$y_{k}={\left[\begin{array}{c} \begin{array}{c} {a_{m}}_{1}\\ {a_{m}}_{2}\\ \vdots \\ {a_{m}}_{n}\\ {\omega_{m}}_{1}\\ {\omega_{m}}_{2}\\ \vdots \\ {\omega_{m}}_{n} \end{array} \end{array}\right]}_{k+1}=h\left(x_{k}\right)=\left[\begin{array}{c} a_{e}+{\dot{\omega }}_{e}\times {s_{sb}}_{1}+\omega_{e}\times \left(\omega_{e}\times {s_{sb}}_{1}\right)+{b_{a}}_{1}\\ a_{e}+{\dot{\omega }}_{e}\times {s_{sb}}_{2}+\omega_{e}\times \left(\omega_{e}\times {s_{sb}}_{2}\right)+{b_{a}}_{2}\\ \vdots \\ a_{e}+{\dot{\omega }}_{e}\times {s_{sb}}_{n}+\omega_{e}\times \left(\omega_{e}\times {s_{sb}}_{n}\right)+{b_{a}}_{n}\\ \omega_{e}+{b_{\omega }}_{1}\\ \omega_{e}+{b_{\omega }}_{2}\\ \vdots \\ \omega_{e}+{b_{\omega }}_{n} \end{array}\right]+{\left[\begin{array}{c} {w_{a}}_{1}\\ {w_{a}}_{2}\\ \vdots \\ {w_{a}}_{n}\\ {w_{\omega }}_{1}\\ {w_{\omega }}_{2}\\ \vdots \\ {w_{\omega }}_{n} \end{array}\right]}_{k}$$

The Kalman filter state propagation matrix is the identity matrix of Equation (2), while its output matrix is the Jacobian of Equation (3):

(4) $$C_{k}={\frac{\partial h}{\partial x}|}_{k}=\left[\begin{array}{cccccccccc} I_{3} & I_{3} & 0_{3} & \cdots & 0_{3} & a_{\omega_{1}} & 0_{3} & 0_{3} & \cdots & 0_{3}\\ I_{3} & 0_{3} & I_{3} & \cdots & 0_{3} & a_{\omega_{2}} & 0_{3} & 0_{3} & \cdots & 0_{3}\\ \vdots & \vdots & \vdots & \ddots & \vdots & \vdots & \vdots & \vdots & \ddots & \vdots \\ I_{3} & 0_{3} & 0_{3} & \cdots & I_{3} & a_{\omega_{n}} & I_{3} & 0_{3} & \cdots & 0_{3}\\ 0_{3} & 0_{3} & 0_{3} & \cdots & 0_{3} & I_{3} & I_{3} & 0_{3} & \cdots & 0_{3}\\ 0_{3} & 0_{3} & 0_{3} & \cdots & 0_{3} & I_{3} & 0_{3} & I_{3} & \cdots & 0_{3}\\ \vdots & \vdots & \vdots & \ddots & \vdots & \vdots & \vdots & \cdots & \ddots & 0_{3}\\ 0_{3} & 0_{3} & 0_{3} & \cdots & 0_{3} & I_{3} & 0_{3} & 0_{3} & \cdots & I_{3} \end{array}\right]$$

where the $a_{\omega_{i}}$ matrix is the Jacobian of the *i*th accelerometer measurements with respect to the attitude rate estimates:

(5) $${a_{\omega }}_{i}=\left[\begin{array}{ccc} q_{e}{s_{y}}_{i}+r_{e}{s_{z}}_{i} & p_{e}{s_{y}}_{i}-2q_{e}{s_{x}}_{i} & p_{e}{s_{z}}_{i}-2r_{e}{s_{x}}_{i}\\ q_{e}{s_{x}}_{i}-2p_{e}{s_{y}}_{i} & p_{e}{s_{x}}_{i}+r_{e}{s_{z}}_{i} & q_{e}{s_{z}}_{i}-2r_{e}{s_{y}}_{i}\\ r_{e}{s_{x}}_{i}-2p_{e}{s_{z}}_{i} & r_{e}{s_{y}}_{i}-2q_{e}{s_{z}}_{i} & p_{e}{s_{x}}_{i}+q_{e}{s_{y}}_{i} \end{array}\right]$$

where $p_{e}$, $q_{e}$ and $r_{e}$, the components of $\omega_{e}$, are the estimates of, respectively, the roll, pitch and yaw rates, and ${s_{x}}_{i}$, ${s_{y}}_{i}$ and ${s_{z}}_{i}$, the components of ${s_{sb}}_{i}$, are the locations of the ith accelerometer along, respectively, the body frame x, y and z axes, with respect to the body centre of mass.

Assuming that all sensors have the same specifications, the state noise covariance matrix (Q) and the measurement noise matrix (R) of the Kalman filter are:

(6) $$Q=\left[\begin{array}{cccccccc} \sigma_{ae}^{2} & 0_{37} & \cdots & 0_{3} & 0_{3} & 0_{3} & \cdots & 0_{3}\\ 0_{3} & {\sigma_{ba}^{2}}_{1}I_{3} & \cdots & 0_{3} & 0_{3} & 0_{3} & \cdots & 0_{3}\\ \vdots & \vdots & \ddots & \vdots & \vdots & \vdots & \ddots & \vdots \\ 0_{3} & 0_{3} & \cdots & {\sigma_{ba}^{2}}_{n}I_{3} & 0_{3} & 0_{3} & \cdots & 0_{3}\\ 0_{3} & 0_{3} & \cdots & 0_{3} & \sigma_{\omega e}^{2} & 0_{3} & \cdots & 0_{3}\\ 0_{3} & 0_{3} & \cdots & 0_{3} & 0_{3} & {\sigma_{b\omega }^{2}}_{1}I_{3} & \cdots & 0_{3}\\ \vdots & \vdots & \ddots & \vdots & \vdots & \vdots & \ddots & \vdots \\ 0_{3} & 0_{3} & \cdots & 0_{3} & 0_{3} & 0_{3} & \cdots & {\sigma_{b\omega }^{2}}_{n}I_{3} \end{array}\right]$$

(7) $$R=\left[\begin{array}{cccccc} {\sigma_{wa}^{2}}_{1}I_{3} & \cdots & 0_{3} & 0_{3} & \cdots & 0_{3}\\ \vdots & \ddots & \vdots & \vdots & \ddots & \vdots \\ 0_{3} & \cdots & {\sigma_{wa}^{2}}_{n}I_{3} & 0_{3} & \cdots & 0_{3}\\ 0_{3} & \cdots & 0_{3} & {\sigma_{w\omega }^{2}}_{1}I_{3} & \cdots & 0_{3}\\ \vdots & \ddots & \vdots & \vdots & \ddots & \vdots \\ 0_{3} & \cdots & 0_{3} & 0_{3} & \cdots & {\sigma_{w\omega }^{2}}_{n}I_{3} \end{array}\right]$$

where ${\sigma_{ba}^{2}}_{i}$ is the variance of the *i*th accelerometer bias, ${\sigma_{b\omega }^{2}}_{i}$ is the variance of the *i*th gyroscope bias, ${\sigma_{wa}^{2}}_{i}$ is the variance of the *i*th accelerometer random walk and ${\sigma_{w\omega }^{2}}_{i}$ is the variance of the *i*th gyroscope random walk. The matrices $\sigma_{ae}^{2}$ and $\sigma_{\omega e}^{2}$ contain the variances of the jerk and angular acceleration, respectively. The variances of the three axes are expressed, individually, along the diagonals of the matrices. The variances of the jerk and of the angular acceleration are estimated based on the expected movements of the sensor, by computing the maximum variation in the expected consecutive measurements. Artillery munitions trajectory is predictable and a good estimation of this variance is achievable. However, for vehicles having unpredictable trajectory (e.g., missiles), the estimation would not be as good.

On their parts, the bias and random walk variances are set to mimic the sensor specifications. As the same sensors are used in all IMUs, all the accelerometers random walk variances $\left({\sigma_{wa}^{2}}_{1\ldots n}\right)$ are equal. This is also the case for the accelerometers bias variances $\left({\sigma_{ba}^{2}}_{1\ldots n}\right)$, for the gyroscopes random walk variances $\left({\sigma_{w\omega }^{2}}_{1\ldots n}\right)$ and for the gyroscopes bias variances $\left({\sigma_{b\omega }^{2}}_{1\ldots n}\right)$. 

As they cannot be measured, the angular accelerations $\left(\dot{\omega }\right)$ must be estimated. This can be done by additional components in the Kalman filter state, or by backward finite differences (Equation (8)). The latter is selected in order to decrease the size of the filter state, hence the computational time:

(8) $${{\dot{\omega }}_{e}}_{k}=\left[\begin{array}{c} {\dot{p}}_{e}\\ {\dot{q}}_{e}\\ {\dot{r}}_{e} \end{array}\right]=\frac{1}{\delta_{t}}\left[\begin{array}{c} {p_{e}}_{k-1}-{p_{e}}_{k-2}\\ {q_{e}}_{k-1}-{q_{e}}_{k-2}\\ {r_{e}}_{k-1}-{r_{e}}_{k-2} \end{array}\right]$$

For algorithm not considering sensors location, the fictitious forces are null. The term ${\dot{\omega }}_{e}\times {s_{sb}}_{i}+\omega_{e}\times \left(\omega_{e}\times {s_{sb}}_{i}\right)$ in Equation (3) and the $a_{\omega_{i}}$ matrix in Equation (4) are then null. There is therefore no relation between the accelerometer and gyroscope measurements, and no coupling between the axes. Then, in order to diminish the computational time, all axes and sensor types are processed separately. The algorithm not considering sensors location is therefore split in three data fusion filters for the acceleration estimation and three others for the attitude rate estimation. The model of each filter is a subset of the complete model (Equations (2) and (3)); it corresponds to the rows and lines of the estimated parameter and the related sensors bias. The model is then:

(9) $$x_{k+1}={\left[\begin{array}{c} a_{e}\\ {b_{a}}_{1}\\ \begin{array}{c} {b_{a}}_{2}\\ \vdots \\ {b_{a}}_{n} \end{array} \end{array}\right]}_{k+1}=\left[\begin{array}{ccccc} 1 & 0 & 0 & \cdots & 0\\ 0 & 1 & 0 & \cdots & 0\\ 0 & 0 & 1 & \cdots & 0\\ \vdots & \vdots & \vdots & \ddots & \vdots \\ 0 & 0 & 0 & \cdots & 1 \end{array}\right]x_{k}+{\left[\begin{array}{c} \begin{array}{c} v_{a}\\ {v_{ba}}_{1} \end{array}\\ {v_{ba}}_{2}\\ \begin{array}{c} \vdots \\ {v_{ba}}_{n} \end{array} \end{array}\right]}_{k}$$

(10) $$y_{k}={\left[\begin{array}{c} {a_{m}}_{1}\\ {a_{m}}_{2}\\ \begin{array}{c} \vdots \\ {a_{m}}_{n} \end{array} \end{array}\right]}_{k}=\left[\begin{array}{ccccc} 1 & 1 & 0 & \cdots & 0\\ 1 & 0 & 1 & \cdots & 0\\ \vdots & \vdots & \vdots & \ddots & \vdots \\ 1 & 0 & 0 & \cdots & 1 \end{array}\right]x_{k}+{\left[\begin{array}{c} {w_{a}}_{1}\\ {w_{a}}_{2}\\ \begin{array}{c} \vdots \\ {w_{a}}_{n} \end{array} \end{array}\right]}_{k}$$

and the covariance matrices are:

(11) $$Q=\left[\begin{array}{ccccc} \sigma_{ae}^{2} & 0 & 0 & \cdots & 0\\ 0 & \sigma_{ba_{1}}^{2} & 0 & \cdots & 0\\ 0 & 0 & \sigma_{ba_{2}}^{2} & \cdots & 0\\ \vdots & \vdots & \vdots & \ddots & \vdots \\ 0 & 0 & 0 & \cdots & \sigma_{ba_{n}}^{2} \end{array}\right]$$

(12) $$R=\left[\begin{array}{cccc} \sigma_{wa_{1}}^{2} & 0 & \cdots & 0\\ 0 & \sigma_{wa_{2}}^{2} & \cdots & 0\\ \vdots & \vdots & \ddots & \vdots \\ 0 & 0 & \cdots & \sigma_{wa_{n}}^{2} \end{array}\right]$$

The previous equations are for the accelerometers. The processing of the gyroscopes is identical, but with the accelerations being replaced by the attitude rates and their corresponding standard deviations. The models of the six filters are then identical. The only difference resides in the values within the covariance matrices, which are related to the estimated parameter.

In the filters initial state, the acceleration and attitude rate estimates are set based on the expected launching state, and the sensors bias are set at 0. On its part, the initial error covariance matrix is set equal to the state noise matrix.

### 2.2. Distributed Architecture

For the distributed architecture, the state of each local filter is composed of 12 elements:

(13) $$x={\left[\begin{array}{cccc} a_{e} & \omega_{e} & b_{a} & b_{\omega } \end{array}\right]}^{T}$$

The state equation is therefore:

(14) $$x_{k+1}={\left[\begin{array}{c} a_{e}\\ \omega_{e}\\ b_{a}\\ b_{\omega } \end{array}\right]}_{k+1}=\left[\begin{array}{cccc} I_{3} & 0_{3} & 0_{3} & 0_{3}\\ 0_{3} & I_{3} & 0_{3} & 0_{3}\\ 0_{3} & 0_{3} & I_{3} & 0_{3}\\ 0_{3} & 0_{3} & 0_{3} & I_{3} \end{array}\right]x_{k}+{\left[\begin{array}{c} v_{a}\\ v_{\omega }\\ v_{ba}\\ v_{b\omega } \end{array}\right]}_{k}$$

while the output equation is:

(15) $$y_{k}={\left[\begin{array}{c} a_{m}\\ \omega_{m} \end{array}\;\right]}_{k+1}=h\left(x_{k}\right)=\left[\begin{array}{c} a_{e}+{\dot{\omega }}_{e}\times s_{sb}+\omega_{e}\times \left(\omega_{e}\times s_{sb}\right)+b_{a}\\ \omega_{e}+b_{\omega } \end{array}\right]+{\left[\begin{array}{c} w_{a}\\ w_{\omega } \end{array}\right]}_{k}$$

A Kalman filter is implemented with its output matrix being the Jacobian of Equation (15):

(16) $$C_{k}={\frac{\partial h}{\partial x}|}_{k}=\left[\begin{array}{cccc} I_{3} & a_{\omega } & I_{3} & 0_{3}\\ 0_{3} & I_{3} & 0_{3} & I_{3} \end{array}\right]$$

Assuming that all sensors have the same specifications, its covariance matrices are:

(17) $$Q=\left[\begin{array}{cccc} \sigma_{ae}^{2} & 0_{3} & 0_{3} & 0_{3}\\ 0_{3} & \sigma_{\omega e}^{2} & 0_{3} & 0_{3}\\ 0_{3} & 0_{3} & \sigma_{ba}^{2}I_{3} & 0_{3}\\ 0_{3} & 0_{3} & 0_{3} & \sigma_{b\omega }^{2}I_{3} \end{array}\right]$$

(18) $$R=\left[\begin{array}{cc} \sigma_{wa}^{2}I_{3} & 0_{3}\\ 0_{3} & \sigma_{w\omega }^{2}I_{3} \end{array}\right]$$

As for the centralized architecture, the angular accelerations cannot be measured and are estimated using backward finite differences (Equation (8)).

For the algorithm not considering sensors location, as for the centralized architecture, the fictitious forces are null and, for each sensor, the data fusion process can be separated in six filters handling the sensor types and axes individually. When the sensors location is not considered, the subset model used in each filter is composed of the rows and lines of Equations (14)–(16) corresponding to the estimated parameter and its related sensor bias:

(19) $$x_{k+1}={\left[\begin{array}{c} a_{e}\\ b_{a} \end{array}\right]}_{k+1}=\left[\begin{array}{cc} 1 & 0\\ 0 & 1 \end{array}\right]x_{k}+{\left[\begin{array}{c} v_{a}\\ v_{ba} \end{array}\right]}_{k}$$

(20) $$y_{k}={a_{m}}_{k}=\left[\begin{array}{cc} 1 & 1 \end{array}\right]x_{k}+{w_{a}}_{k}$$

and the covariance matrices are:

(21) $$Q=\left[\begin{array}{cc} \sigma_{ae}^{2} & 0\\ 0 & \sigma_{ba}^{2} \end{array}\right]$$

(22) $$R=\sigma_{wa}^{2}$$

The previous equations are for the accelerometers. The processing of the gyroscopes is identical, but with the accelerations being replaced by the attitude rates and their corresponding standard deviations.

The state of each local filter is initialized based on the expected launching state. The sensors bias are set at 0, and each local filter error covariance matrix is set identical to the state noise matrix.

For both cases, considering or not the sensors location, following the local filters computation, a master data fusion algorithm computes the mean of all estimations for each parameter. Those means are the final estimates, which are sent to the local filters to reset their estimates before the next time step.

### 2.3. Baseline Architecture

In order to better assess the performance of two previous architectures, a baseline architecture is proposed. The baseline architecture computes for each axis (*x*, *y* and *z*) and sensor type (gyroscope and accelerometer), the measurements average. However, because of the centrifugal and angular accelerations, pre-computations are required on the accelerometer measurements before computing their means.

First, the angular velocity is obtained by directly computing, on each axis separately, the mean of the gyroscope measurements.

The acceleration and attitude rate estimations are then used to remove the angular and centrifugal accelerations from the accelerometer measurements:

(23) $$a_{i}={a_{m}}_{i}-\;\left({\dot{\omega }}_{e}\times s_{sb}\right)-\left(\omega_{e}\times \left(\omega_{e}\times s_{sb}\right)\right)$$

As previously, the angular accelerations, required in the linear accelerations computation, are estimated by backward finite differences (Equation (8)).

The acceleration estimates are the means of the Equation (23) subtractions:

(24) $${a_{x}}_{e}=\frac{\sum_{i=1}^{n}\left({a_{mx}}_{i}-\left(p_{e}q_{e}{s_{y}}_{i}-q_{e}^{2}{s_{x}}_{i}+p_{e}r_{e}{s_{z}}_{i}-r_{e}^{2}{s_{x}}_{i}\right)-\left({\dot{q}}_{e}{s_{z}}_{i}-{\dot{r}}_{e}{s_{y}}_{i}\right)\right)}{n}$$

(25) $${a_{y}}_{e}=\frac{\sum_{i=1}^{n}\left(a_{{my}_{i}}-\left(p_{e}q_{e}{s_{x}}_{i}-p_{e}^{2}{s_{y}}_{i}+q_{e}r_{e}{s_{z}}_{i}-r_{e}^{2}{s_{y}}_{i}\right)-\left({\dot{r}}_{e}{s_{x}}_{i}-{\dot{p}}_{e}{s_{z}}_{i}\right)\right)}{n}$$

(26) $${a_{z}}_{e}=\frac{\sum_{i=1}^{n}\left({a_{mz}}_{i}-\left(p_{e}r_{e}{s_{x}}_{i}-p_{e}^{2}{s_{z}}_{i}+q_{e}r_{e}{s_{y}}_{i}-q_{e}^{2}{s_{z}}_{i}\right)-\left({\dot{p}}_{e}{s_{y}}_{i}-{\dot{q}}_{e}{s_{x}}_{i}\right)\right)}{n}$$

For the algorithm not considering sensors location, the fictitious forces terms can be removed from the previous equations. The acceleration estimates then become the average, on each axis separately, of the accelerometers measurements only.

## 3. Fault Detection and Isolation Algorithm

The FDI algorithm, identical for the three architectures, is based on a statistical analysis of the residual. There are systematic ways to deal with faulty sensors [6,25], but as this work realizes relative comparisons of data fusion architectures, a simple FDI method is used.

The residual is the difference between the sensor measurement, and the parameter and sensor bias estimates:

(27) $$r_{ax_{1}}={a_{x}}_{1}-\left({a_{x}}_{e}+b_{ax_{1}}\right)$$

For the baseline architecture, where the bias is not estimated, the latter is considered null in the residual computation.

If this residual is larger than four times the standard deviation of the sensor random walk for two consecutive measurements, the sensor is considered faulty. The two consecutive 4σ threshold is selected in order to limit false warnings, while ensuring detection of faulty sensors. The sensor random walk being considered as a normal distribution, there is 0.0063% (1 in 15,787) chance that a healthy sensor gives a value larger than the threshold. With a sample time of 0.001 s, this is a false warning every 16 s. For two consecutive measurements, this percentage drops to 4.01 × 10^−9^% (1 in 249,229,369), which is approximately a false warning every 29 days. This percentage is equivalent to a single value larger than a threshold of 5.9*σ*, approximately. However, because of the exponential tail of the normal distribution, a faulty sensor, which is a sensor with a random walk of standard deviation larger than the nominal sensor, has more chance of producing two consecutive measurements larger than 4*σ* than a single measurement larger than 5.9*σ*.

## 4. Performance Analysis

The architectures and algorithms are tested, in simulation, on a spin-stabilized projectile. A typical low quadrant elevation, low muzzle velocity and Northward launch trajectory is obtained from a projectile simulator developed in a non-spinning body frame [23]. The sensors measurements are simulated by adding random noises and bias instabilities to the nominal accelerations and attitude rates. Except when otherwise mentioned, the noises and biases are completely uncorrelated.

Two series of tests are done. First, the quality of the estimation of each architecture and algorithm, without faulty sensors, is tested (Section 4.1). Then, the performance of the fault detection algorithm is analysed (Section 4.2). For both series, the sensors are distributed around the projectile centre of mass. Hence, each accelerometer measures a different acceleration based on the sensed fictitious forces at its location.

### 4.1. Estimation Precision and Accuracy

As non-collocated sensors are used, the distance between the IMUs, their relative locations and their numbers influence the quality of the estimation of each algorithm. These three parameters are separately analysed.

#### 4.1.1. Relative Locations of the IMUs

**Figure 1:**
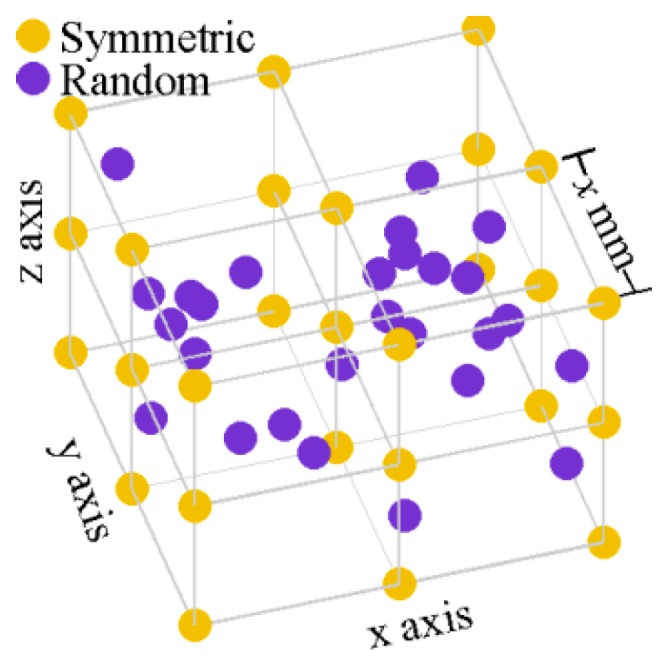
Symmetric and random 27 IMUs configurations.

The effects of the relative locations of the IMUs are studied by comparing a symmetric 27 IMUs configuration to a configuration where the 27 IMUs are randomly located. In the random configuration, the sensors are all located within the cube of the symmetric configuration (Figure 1) and the randomly chosen positions are kept for all simulations. The random configuration is physically impracticable, but it is interesting to evaluate the sensors dissymmetry effect on the algorithms.

Monte-Carlo simulations, composed of 350 runs each, are executed for 10 and 100 mm separations between the symmetric IMUs (20 and 200 mm edges cubes), and for their corresponding random locations. As the gyroscope measurements are not affected by their locations, the estimation of the attitude rate is not affected by the random configuration. However, the quality of the acceleration estimation decreases with the random configuration. Table 1 and Table 2 show the standard deviation of the acceleration estimation errors.

As expected, without symmetry in the measured fictitious accelerations, the three algorithms not considering sensors location provide completely unusable results. On their part, the algorithms considering sensors location are differently affected by the random configuration. For the three architectures, the performance degradation is larger for the 200 mm edges cube than for the 20 mm one. Also, for the 200 mm edges cube, the degradation is much larger for the baseline architecture than for the two others. The knowledge of the sensor noises characteristics, which is introduced into the Kalman filters, helps at conserving the quality of the estimation with the centralized and distributed architectures. When the locations are considered, the main reason to explain the performances degradation due to random configuration is the sensors bias estimation. The centralized architecture provides a better estimation of the biases (Section 4.3) than the distributed architecture. It is, therefore, less affected by the random configuration. Both architectures use the redundancy and apparent observability differently; the centralized architecture uses it to improve the estimation of the biases, while the distributed architecture uses it to improve the acceleration and attitude rate estimations.

#### 4.1.2. Relative Distance between the IMUs

The distance between 27 IMUs is varied from 1 mm to 100 mm. A symmetric 27 IMUs configuration, a cube with nine IMUs evenly distributed on each face and one IMU in the middle of the cube, at the centre of mass, is selected (Figure 1). This configuration creates symmetries in the sensed fictitious accelerations.

**Figure 2:**
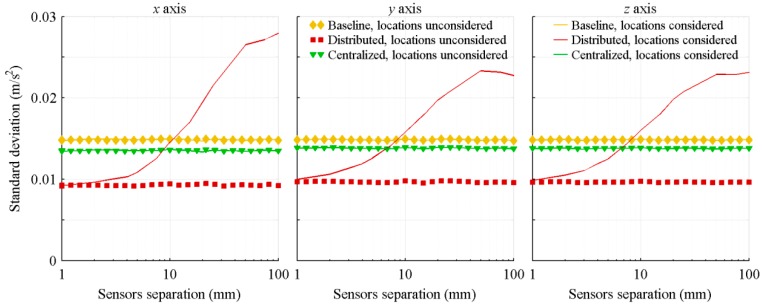
Standard deviation of the acceleration estimation errors, with respect to the distance between the IMUs.

**Figure 3:**
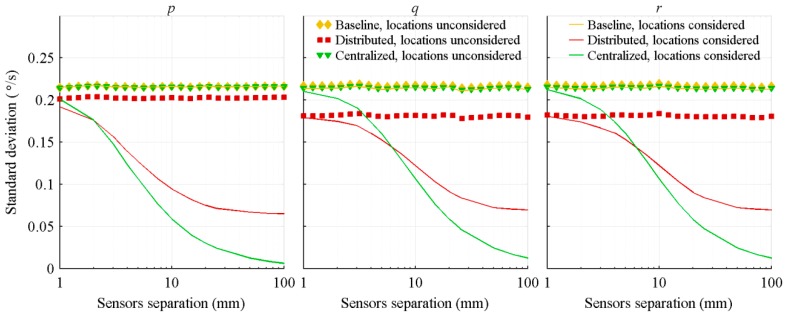
Standard deviation of the attitude rate estimation errors, with respect to the distance between the IMUs.

Figure 2 presents the resulting standard deviation of the acceleration estimation errors and Figure 3 shows the standard deviation of the attitude rate estimation errors. Those results are obtained with a Monte-Carlo simulation composed of 350 runs for each sensor separation. For each run covering the full projectile trajectory, the standard deviation of the estimation errors is computed. The resulting standard deviations are then averaged over the 350 runs, and the result presented as a function of the sensors separation.

As a symmetric sensors configuration is used, the algorithms not considering sensors location are still very efficient. For the acceleration estimation with the centralized and baseline architectures, there is no gain at considering the relative locations of the sensors in the algorithms. The fictitious accelerations sensed by one sensor are always cancelled by the corresponding symmetrical sensor. For the distributed architecture, there is even a loss in efficiency when the sensors location is considered in the algorithm. In this architecture, the acceleration of each IMU is estimated prior to the averaging. Therefore, portions of the fictitious accelerations are considered as real accelerations and are not canceled by the sensors symmetry.

Considering the sensors location in the algorithms significantly improves the precision of the attitude rate estimation. By taking into account the sensors location, the accelerometers are combined with the gyroscopes in the attitude rate estimation. When the sensors separation grows, the fictitious accelerations increase and are more easily discriminated over the accelerations of the sensor centre of mass and noises. The performance improvement is therefore more important for larger separation. Also, the centralized architecture better exploits those fictitious accelerations than the distributed architecture. The first one considers the sensors configuration as a whole, and therefore, simultaneously estimates the fictitious accelerations sensed by all accelerometers. On its part, the distributed architecture treats each IMU separately and does not exploit the sensors configuration as a whole. It only uses the location of each IMU with respect to the centre of mass. Therefore, portions of the fictitious accelerations are considered as real accelerations. The worse results of the distributed architecture, in comparison to the centralized architecture ones, are emphasized by its poor biases estimation (Section 4.3).

Also, due to the nature of the projectile trajectory and to the Kalman filters tuning, the three projectile axes show similar performance. The motions of the projectile along its y and z axes are not significantly different, and are in the same order of magnitude as its motion along its x axis (linear deceleration). The only parameter which is significantly different than the others is the projectile x axis spin rate which decreases rapidly. Therefore, a larger value is given to the variance of the projectile spin rate $\left(\sigma_{p}^{2}\right)$. For the algorithms not considering sensors location, the improvements of the centralized and distributed architectures in comparison to the baseline one are slightly less important for this parameter (left graphic of Figure 3). However, for the algorithms considering sensors location, the improvements are more significant for this parameter than for the others. The nominal spin rate of the projectile being much larger than the two other rates, the fictitious accelerations due to this rate are larger, and therefore, are more easily distinguishable.

#### 4.1.3. Number of Near Symmetrically Located IMUs

As demonstrated in Section 4.1.2, the symmetry of the sensors significantly influences the estimation performance. Therefore, in this analysis, the locations are selected in order to have a close to symmetric distribution for all numbers of IMUs. In this way, the algorithms not considering sensors location can be applied and provide relatively good performance.

Also, a 10 mm separation between the sensors is selected. For close to symmetric distribution, this distance is the larger one allowing the packaging of all sensors within a projectile fuze. Furthermore, based on Section 4.1.2, this distance still provides a significant gain in the attitude rate estimation.

**Figure 4:**
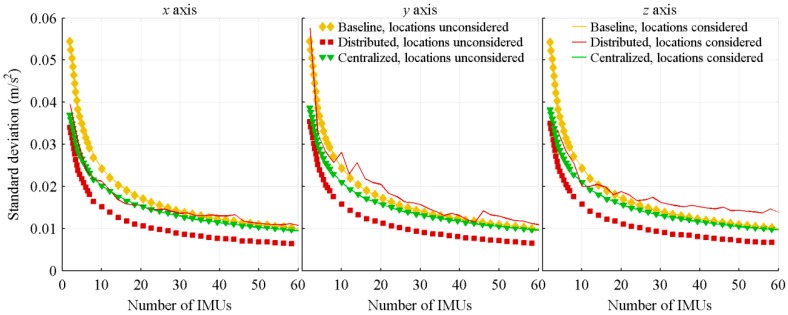
Standard deviation of the acceleration estimation errors, with respect to the number of IMUs.

**Figure 5:**
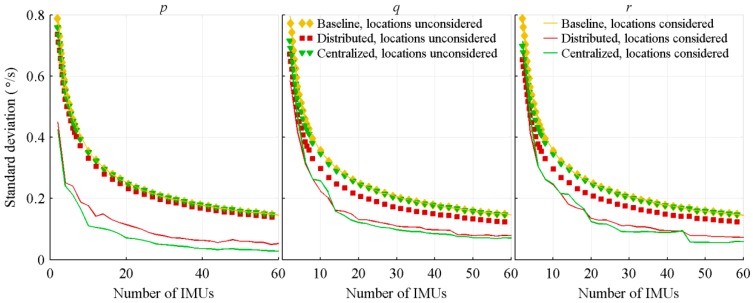
Standard deviation of the attitude rate estimation errors, with respect to the number of IMUs.

A Monte-Carlo simulation composed of 350 runs is then executed for each IMU number, which is varied from 2 to 60 by steps of 2. For each run, covering the full projectile trajectory, the standard deviation of the estimation errors is computed. The resulting standard deviations are then averaged over the 350 runs, and the result presented as a function of the number of IMUs in Figure 4 and Figure 5. 

As expected, a trend coherent with the signal averaging theory, a diminution of the standard deviation proportional to 1n, is obtained for all cases. For the algorithms not considering sensors location, the distributed architecture provides the best performance. Because of the local filtering, the mean of the master filter is computed on data having a smaller standard deviation than the raw measurements, resulting in a less noisy final estimation for the distributed architecture than for the baseline architecture. On its part, because of the covariance matrices tuning which gives equal weight to all measurements, the centralized architecture is, when the sensors location is not considered, similar to directly computing the mean of the measurements. The small performance improvement, in comparison to the baseline architecture, comes from the a priori knowledge of the trajectory and noises introduced into the Kalman filter. The two Kalman filterbased architectures, therefore, use the redundancy differently: the centralized one improves the biases estimation (Section 4.3), while the distributed one improves the parameter estimations.

However, as in Section 4.1.1, due to how it handles the fictitious accelerations, the centralized architecture with its algorithm considering sensors location is the best option for nearly all tested number of IMUs. The light geometric dissymmetry for some numbers of sensors, and the non-linear variation, with respect to the number of IMUs, of the distance between the centre of mass of the IMUs configuration and the farthest IMU, explain the non-smooth behaviour of the distributed architecture acceleration estimation and of the centralized and distributed architectures attitude rate estimation. 

As in Section 4.1.1, the algorithms considering sensors location are, for the attitude rate estimation, better than those not considering them, but the distributed architecture acceleration estimation is worse when the sensors location is considered. Also, the acceleration estimation errors of the centralized and baseline architectures are not significantly modified by the inclusion of the sensors location within the algorithms. As in Section 4.1.1, because of the large nominal value of the spin rate, the estimation of this parameter differs from the others; the centralized and distributed architectures with their algorithms not considering sensors location are slightly less efficient for the spin rate axis than for the other axes, the opposite being observed for those architectures when the sensors location is considered in the algorithms.

All previous results were obtained with fully uncorrelated bias instabilities. However, MEMS are expected to have some correlations between them. Therefore, a second series of tests, with fully correlated biases is done. For these tests, the same bias is applied to all sensors generating measurements for the same parameter. There are no correlations between the axes and, as it is nearly impossible to know the sensors’ correlation in a real system, the knowledge of the correlation is not included in the Kalman filter covariance matrices. 

**Figure 6:**
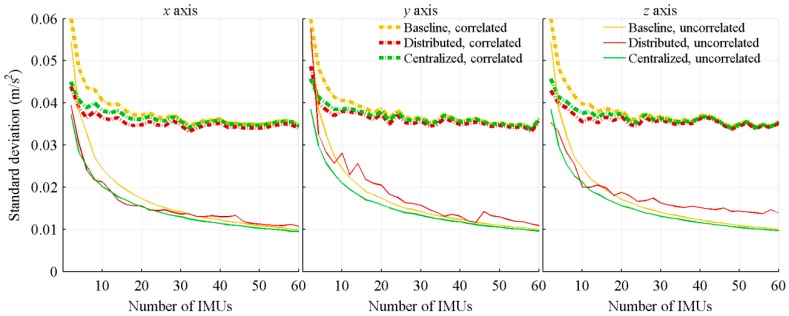
Standard deviation of the acceleration estimation errors, with correlated and uncorrelated sensors bias, and function of the number of IMUs.

**Figure 7:**
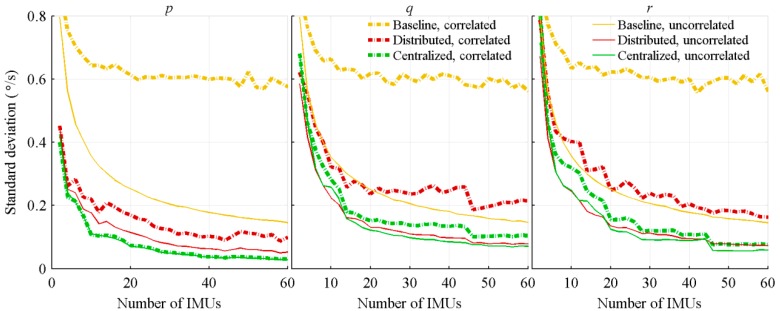
Standard deviation of the attitude rate estimation errors, with correlated and uncorrelated sensors bias, and function of the number of IMUs.

The following graphics compare the attitude rate (Figure 6) and acceleration (Figure 7) estimation errors of the correlated biases tests to the previous uncorrelated ones. Obviously, the biases of the sensors measuring the same parameter are not expected to be fully correlated nor fully uncorrelated. However, comparing the two sets of results provides bounds for the achievable performance. In order to keep the graphics readable, the results of the algorithms considering sensors location were included only. For correlated biases, the three algorithms not considering sensors location provide estimations nearly identical to those of the baseline algorithm considering sensors location.

When the sensors location is considered, the accelerometers provide supplementary information on the attitude rate, allowing the distinction between the biases and vehicle motions. This is the main explanation of the improvements, with respect to the number of sensors, obtained with fully correlated biases. On their parts, the three architectures’ acceleration estimation and the baseline attitude rate estimate provide, as mentioned in the literature [20], small improvements with respect to the number of considered IMUs. The biases cannot be clearly distinguished from the vehicle motions, and increasing the number of IMUs does not fix the issue.

Up to this point, the analysis is focusing on the precision of the estimation. The second important characteristic of the quality of an estimation is its accuracy, characterized by the mean of the estimation errors. For the tested cases, the mean is always near 0 and does not show a specific trend with respect to the number of IMUs.

**Figure 8:**
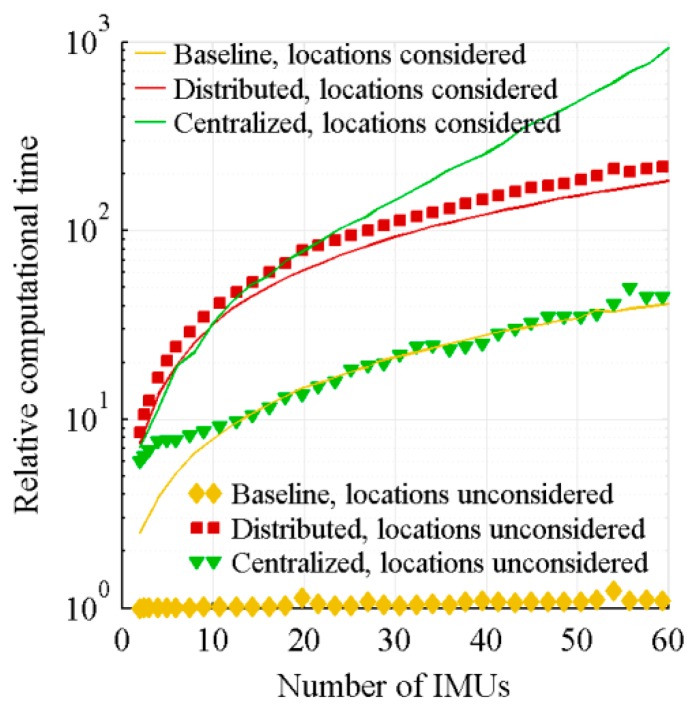
Relative computational time.

The third studied characteristic of the algorithms is the relative computational time, which is presented in Figure 8 as a function of the number of IMUs. As the sensors’ bias correlation does not affect the computational time, only the times of the fully uncorrelated biases tests are presented. The reference computational time is the computational time of the baseline architecture with its algorithm not considering sensors location in the case of two IMUs. Table 3 summarizes the operation that requires the most computational time for each architecture and the number of times this operation must be performed.

For the baseline architecture, the data fusion is implemented through, quickly performed, basic mathematical operations and the computation time variation as a function of the number of IMUs is marginal for the algorithm not considering sensors location. However, for the algorithm considering sensors location, the estimation of the angular acceleration which requires n cross-products is more time consuming and produces an increase of the computational time proportional to the number of sensors.

For the distributed architecture, the computational time increments come from the time required to compute the added local filters. For the algorithm not considering sensors location, each local filter has to compute six divisions, while for the algorithm considering sensors location, it must invert a 6 × 6 square matrix. Both operations are similar in term of computational time, the small difference being rather due to the larger amount of data handling operations for the algorithm not considering sensors location. 

For the centralized architecture, the computational time is driven by the inversion of the Kalman filter covariance matrix. When the sensors location is considered, the addition of an IMU adds six rows and six columns to the matrix to be inverted. However, when the sensors location is not considered, it rather adds one row and one column to each of the six covariance matrices. For a relatively small symmetric matrix, the inversion is quickly performed. Therefore, among the algorithms not considering sensors location, the centralized architecture, which requires less handling operations, is faster than the distributed one. However, for large matrix, typical of the centralized architecture with its algorithm considering sensors location, the computational time is much longer.

The presented data fusion algorithms do not provide attitude, velocity and position estimations, which are provided by a standard INS. Also, without GPS receiver, there is no corrections possibility on these estimations. The estimated attitude rate is therefore directly integrated to estimate the attitude. On its part, the estimated acceleration, which is measured in the body frame, is transposed, by a rotation matrix, in the North-East-Down (NED) frame and integrated, in this frame, to estimate the position and velocity. 

**Figure 9:**
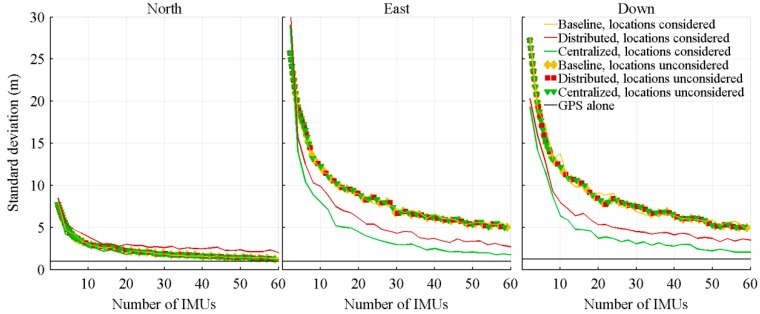
Standard deviation of the position errors when the projectile hits the ground, with respect to the number of IMUs.

The standard deviation of the position errors is shown in Figure 9. As a comparison point, the typical precision of a military grade GPS receiver is included in the graphics.

Even if the precision of the acceleration and attitude rate estimations differ between the algorithms, the standard deviation of the position errors when the projectile hits the ground is not significantly different between the algorithms not considering sensors location, the gaps being not even visible in the graphics. The distributed architecture is better than the two others by, approximately, 0.01 m.

However, for the algorithms considering sensors location, the centralized and distributed architectures offer a significant gain over the baseline architecture, the improvements coming from the better attitude rate estimation. The accelerations are measured in the body frame, while the positions are expressed in the NED coordinates. To migrate from the body frame to the NED frame, the accelerations must be rotated. The rotation is performed by a rotation matrix which is directly affected by the attitude rate estimation. 

### 4.2. Fault Detection

The efficiency of the algorithm proposed for FDI is tested on a specific number of IMUs and a fixed number of faults. The symmetric 27 IMUs configuration of Section 4.1.2, with three faults for the accelerometers and three faults for the gyroscopes, is used to assess the FDI performance of the three architectures equipped with the proposed FDI algorithm. A single form of faulty sensor, a sensor producing a measurement related to the vehicle motion, but with a larger random walk than the sensor typical one, is studied. The faulty sensor, the time of the fault and the amplitude of the faulty random walk are randomly set prior to each simulation. The amplitude of the random walk of the faulty sensor is, at least, 1.75 times that of a healthy sensor, and the fault occurs, at least, 1 s prior to the end of the simulation. 

**Figure 10:**
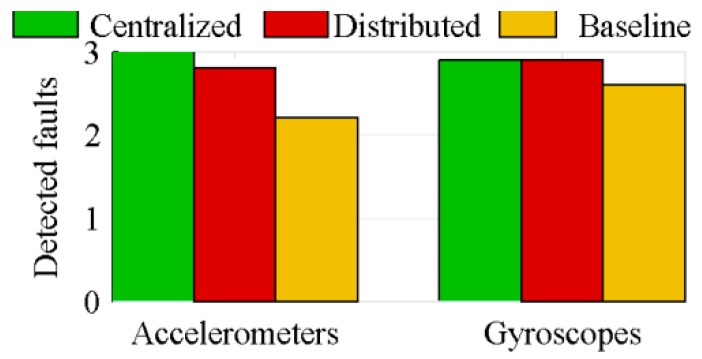
Detected faults for the algorithms considering sensors location.

**Figure 11:**
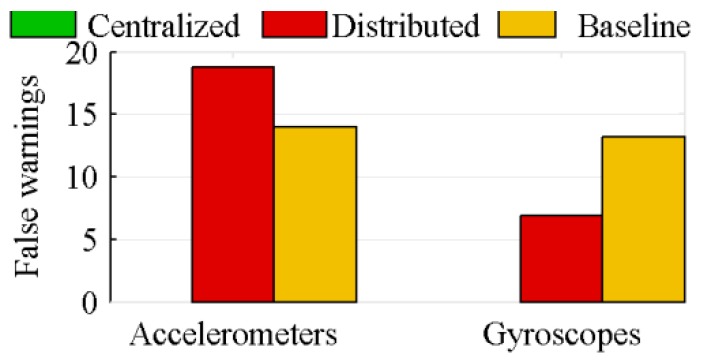
False warnings for the algorithms considering sensors location.

In this section, only the algorithms considering sensors location are studied. The algorithms not considering sensors location quickly consider all accelerometers as faulty, because they assimilate the fictitious accelerations to the sensors bias. Figure 10 and Figure 11, respectively, show the mean numbers of correctly detected faulty sensors and of false warnings, based on Monte-Carlo simulations of 100 runs each.

**Figure 12:**
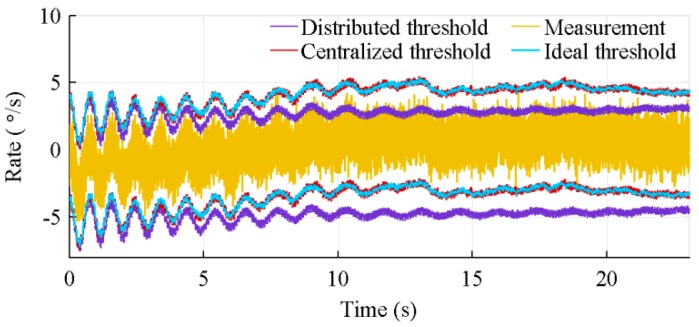
Typical 4σ thresholds of the centralized and distributed architectures with the algorithms considering sensors location, for 27 IMUs.

Because of its inability to correctly estimate the sensors bias, the distributed architecture generates a lot of false warnings, while the centralized architecture correctly identifies nearly all faulty sensors. The effect of this poor biases estimation is shown in Figure 12. This figure presents the 4*σ* thresholds of the centralized and distributed architectures over the measurements of a healthy sensor, when 27 IMUs are used. Because of its drift, at 8.9 s, this healthy sensor is considered faulty by the distributed architecture. The ideal threshold, added to the graphic, is the threshold computed on the nominal biased value. The latter is the measured value without noise.

The bias estimation also explains why there are more false warnings for the accelerometers than for the gyroscopes. Because of the fictitious forces, the measured acceleration magnitude is much larger than the measured attitude rate one. The distributed architecture has difficulty to estimate the sensors bias in presence large measured values (Section 4.3).

**Figure 13:**
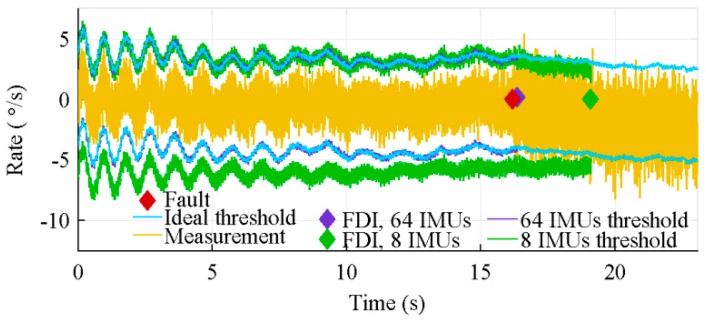
Typical 4σ threshold of the centralized architecture with the algorithm considering sensors location, for 8 and 64 IMUs.

The proposed FDI algorithm is therefore dependent of the number of IMUs. With more IMUs, the standard deviation of the residual decreases and the bias estimation improves. A smaller standard deviation smoothes the threshold bounds, while a bias estimation improvement tightens them. Figure 13 shows, for 8 and 64 IMUs, the threshold of the centralized architecture and the detection time of a faulty sensor. Because of a better threshold estimation, the fault is detected nearly 6 s earlier with 64 IMUs. 

### 4.3. Bias Estimation

Most of the results obtained previously are highly influenced by the inclusion of the sensors bias estimation within the centralized and distributed architectures. An analysis of this bias estimation is therefore done.

**Figure 14:**
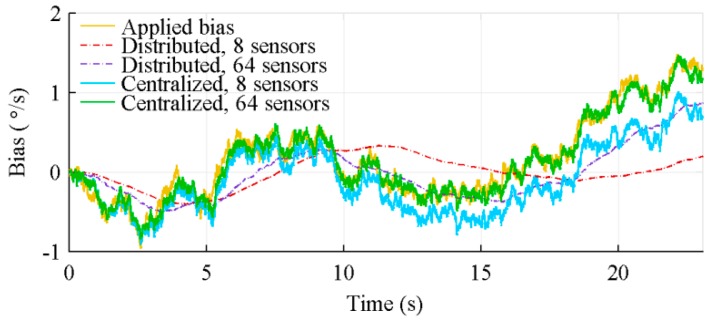
Bias estimation of gyroscopes measuring p, for 8 and 64 IMUs, with algorithms considering sensors location.

Both, the distributed and centralized architectures, have an unobservable subspace. However, they both show the typical behaviour of a fully observable system. Notably, they are able to come back to the true value after a significant outlier. This apparent observability comes from the correlation between the measurements, which is used to extract supplementary information for the sensors bias estimation. The centralized architecture is however better at estimating those biases, as shown in Figure 14, which shows the estimation of a bias on a gyroscope measuring the spin rate in a 8 and a 64 sensors configuration, the sensors location being considered. Furthermore, also visible on Figure 14, with more sensors, which favour the extraction of supplementary information, the biases estimation is improved.

**Figure 15:**
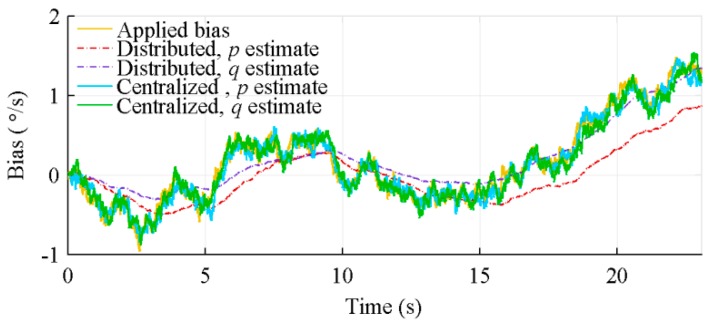
Bias estimation of gyroscopes measuring p and q, for 64 IMUs, with algorithms considering sensors location.

Figure 15 illustrates the difficulty of the distributed architecture, due to the a priori knowledge of the trajectory (sensor movements) introduced into the Kalman filter state noise covariance matrix, to estimate the sensors bias when the expected measurement variation is large. In this figure, the same bias is applied on gyroscopes measuring p (large value) and q (small value). This figure shows that the centralized architecture bias estimation is marginally affected by the amplitude of the measurements, while the distributed architecture one is significantly better for q.

## 5. Conclusions

This work has analysed two data fusion architectures, a centralized architecture and a distributed architecture, to treat the measurements of a large amount of orthogonal redundant inertial measurement units. For each architecture, an algorithm considering the location of the sensors and another one not considering it have been proposed. In the centralized architecture, all sensor measurements are simultaneously processed by a single Kalman filter. In the distributed architecture, the measurements of each sensor are filtered by a local Kalman filter before being sent to a master data fusion filter which computes the final estimate. A baseline architecture, which directly computes the mean of the measurements, is used as a point of comparison. As this work focused on Micro-Electronic-Mechanical Systems, whose one of their main weaknesses is their large bias instability, the implemented Kalman filters were designed to estimate the sensors bias.

The Monte-Carlo simulations demonstrated that the centralized architecture with its algorithm considering sensors location provides the best performance. Its estimations of the accelerations, attitude rates and sensors bias are the most precise and accurate. It is also less affected when the IMUs are randomly located and its acceleration estimation does not degrade when the distance between the IMUs is increased. Additionally, it provides better fault detection capabilities, detecting nearly all faults and generating few false warnings. However, its better performance comes at the expense of a much longer computational time. The distributed architecture with its algorithm considering sensors location provides, at a much lower computational time, good estimations of the accelerations and attitude rates. However, the quality of these estimations degrades when the IMUs are located randomly, and its fault detection capabilities are very limited. The centralized and distributed architectures with their algorithms not considering sensors location remain viable for nearly symmetric locations, but they are completely unusable when the IMUs are located randomly, and for fault detection. With correlated sensors bias, only the centralized and distributed architectures with their algorithms considering sensors location are able to obtain a significant improvement, with respect to the number of IMUs, of the attitude rate estimation. Also, in presence of sensors bias correlation, no matter which architecture is used, the quality of the acceleration estimation is barely modified by the number of sensors.

The results and analyses of this paper were obtained under perfect conditions for the filtering algorithms, which means that the latter were always set with perfect knowledge of the sensors’ characteristics and their locations. This study could be extended to cases considering an approximate knowledge of this information. Also, a more in-depth analysis, involving real flight data with a specific number of IMUs, would be done to validate the simulation results.

## Figures and Tables

**Table 1:** Standard deviation of the acceleration estimation errors, for the symmetric and random 27 IMUs configurations within the 20 mm edges cube.

IMUs Distribution	Axis	Locations Considered (m/s^2^)			Locations Unconsidered (m/s^2^)		
		Centralized	Distributed	Baseline	Centralized	Distributed	Baseline
Symmetric	x axis	0.0136	0.0146	0.0150	0.0136	0.0094	0.0150
	y axis	0.0139	0.0158	0.0149	0.0139	0.0098	0.0149
	z axis	0.0139	0.0160	0.0149	0.0139	0.0097	0.0149
Random	x axis	0.0142	0.0150	0.0171	0.0158	0.0124	0.0169
	y axis	0.0142	0.0174	0.0173	16.01	16.11	16.01
	z axis	0.0142	0.0173	0.0174	22.93	23.08	22.93

**Table 2:** Standard deviation of the acceleration estimation errors, for the symmetric and random 27 IMUs configurations within the 200 mm edges cube.

IMUs Distribution	Axis	Locations Considered (m/s^2^)			Locations Unconsidered (m/s^2^)		
		Centralized	Distributed	Baseline	Centralized	Distributed	Baseline
Symmetric	x axis	0.0135	0.0279	0.0148	0.0135	0.0092	0.0148
	y axis	0.0137	0.0227	0.0148	0.0137	0.0096	0.0148
	z axis	0.0138	0.0231	0.0148	0.0138	0.0096	0.0148
Random	x axis	0.0151	0.0377	0.0876	0.0839	0.0833	0.0842
	y axis	0.0144	0.0416	0.0910	160.14	161.12	160.16
	z axis	0.0143	0.0469	0.0914	229.29	230.75	229.32

**Table 3:** Most time consuming operation for each architecture.

Architecture	Locations Considered	Locations Unconsidered
Centralized	Invert a 6n×6n matrix	Invert 6 n×n matrices
Distributed	Invert n 6 × 6 matrices	Compute 6n divisions
Baseline	Compute 3n cross-products	Mean computations
